# HIV in Pakistan: Challenges, efforts and recommendations

**DOI:** 10.1016/j.amsu.2022.104797

**Published:** 2022-11-02

**Authors:** Yumna Salman, Sean Kaisser Shaeen, Malaika Saeed Butt, Laiba Imran Vohra, Taleen Hashmi

**Affiliations:** Faculty of Medicine, Dow Medical College, Dow University of Health Sciences, Pakistan; Department of Medicine, Ziauddin University, Pakistan; Faculty of Medicine, Dow Medical College, Dow University of Health Sciences, Pakistan

According to the World Health Organization (WHO), the Human Immunodeficiency Virus (HIV) is an RNA retrovirus which causes an infection by suppressing the body's immune system [1]. It does so primarily by depleting important disease fighting immune cells, specifically CD4^+^ helper T-cells, resulting in host susceptibility to opportunistic infections.

Such as tuberculosis and pneumonia [[2], [3], [4]]. HIV has main types alongside their variable sub-strains, with HIV-1 being the most transmissible and aggressive one of the two [5,6]. HIV-1 is the most common, identified in Pakistan and throughout most of the world, accounting for around 95% of all cases, while HIV-2 is a milder form mainly limited to West Africa [7].

Transmission varies across many routes, including but not limited to exposure to HIV infected bodily fluids via sexual contact, blood transfusions and IV abuse [8]. Vertical transmission, during delivery or breastfeeding, is another possible opportunity for the spread of HIV [8]. The presentation in this condition can vary from being asymptomatic to symptoms usually being defined as that of a “flu-like illness” depending on the stage of the infection [9].

The pathogenesis of HIV typically consists of three stages: the acute stage 1, chronic stage 2, and advanced stage 3 [9]. Stage-1 is the early stage that usually present with symptoms of flu-like illness and is relatively contagious [9]. Stage-2 is the asymptomatic clinically latent phase of the infection, medical therapy should be followed to ensure this stage does not progress to stage-3, the advanced stage also known aacquired immunodeficiency syndrome (AIDS), which is extremely infectious and easily transmissible due to the high viral load by this point [9]. If the proper diagnosis along with proper compliance with the Anti Retro-Viral Therapy (ART) is not followed through with as treatment, this condition can progress to a much more severe form known as AIDS (Acquired Immuno-Deficiency Syndrome) [9]. If untreated, HIV typically progresses into AIDS in about 8–10 years on average [10]. This is said to happen when a person with HIV has their CD4 cell count fall below 200 cells per cubic millimeter of blood or has an additional AIDS-defining condition such as Pneumocystis Pneumonia [11].

Pakistan ranks as the 5th most densely populated country in the world, playing a role in its status of being a high-risk but low prevalence when it comes to the degree of HIV spread [12]. Poverty, illiteracy, and unhygienic practices are just some of the factors that account for the high-risk status of HIV in Pakistan [13]. All these high-risk factors contribute to the ongoing stigma around the routine testing of HIV making surveillance and control of this infection an ongoing problem [14]. The low prevalence of HIV in the Pakistani population, approximated to be less than 0.1% by the recent UNIAIDS 2020 progress report, can be accounted for by the religious practice of abstinence from non-marital sexual interaction in Pakistan [15]. The two major provinces of Pakistan, Sindh and Punjab together make up around 91% of the total number of PLHIV (people living with HIV) in this country [15]. Centring in, the major hubs of PLHIV are made up of the most populated cities in Pakistan such as Karachi, Lahore, and Islamabad [15]. Even though a global decline in new HIV cases was seen, there have been an increasing number of outbreaks in Pakistan leading it to be one of the fastest rising HIV nations across Asia [16].

The aim of this paper is to highlight the challenges of HIV in Pakistan and discuss relevant efforts made alongside providing recommendations to help better manage its outbreaks throughout the country.

A large proportion of the Pakistani population is uneducated and is relatively uninformed of the signs and symptoms, underlying causes, and the safety precautions necessary to limit HIV transmission. This leads to its increased rates of transmission and unintentional spread [17]. HIV spreads in people who engage in actions which are likely to increase the odds of contracting it, such as, extramarital relationships, homosexual intercourse, or intravenous (IV) drug usage [18]. Another significant aspect that leads to the increase in cases would be scenarios in which infected individuals conceal their condition and alienate themselves due to the fear of prejudice from their own communities [18]. This prejudice against people living with HIV is adding to the serious threat to prevention and early treatment of the condition and is hampering efforts to curb and address the epidemic considerably [18,19]. Moreover, because of routine blood donation and transfusion procedures, where health care institutions frequently have subpar facilities and practices as well as the neglect of blood screening, there's an increased tendency of HIV becoming more widespread [20]. Although there are legal prostitution establishments in all of Pakistan's major cities, the government is unwilling to acknowledge the unlawful sex that is taking place there, posing an additional risk for HIV transmission [21]. Along with female prostitutes, the districts are home to male transvestites known as “Hijras”, and the fact that most males engaging in male-to-male sexual activity in Pakistan are married, highlights their ability to serve as a conduit to the broader community [21,22]. In 2020, the prevalence of HIV amongst the transgender community was estimated to be 5.5%, making them among the highest risk groups for HIV [23]. Healthcare workers (HCW) are more susceptible to infections because of non-adherence to WHO guidelines, such as careless handling of contaminated needles, reuse of needles that weren't properly sterilized, improper disposal of potentially dangerous waste, and overcrowding in patient care areas, which may occur as a consequence of not having a suitable reporting channel or a suitable process for reporting workplace accidents [24]. Mengistu DA et al. reported a study involving 4235 participants, out of which 2470 (58.3%) HCW were exposed to at least one needle stick injury at work, demonstrating a significant danger of being exposed to numerous blood-borne diseases like HIV [25]. Although the National AIDS Control Program (NACP), with the assistance of the Global Fund, has established 50 HIV treatment centers across various provinces in the nation, where people have to bear great travel costs with long waiting times, there aren't enough expertise in infectious diseases to run ART clinics, and primary care workers lack the knowledge and training necessary to effectively manage HIV-positive patients [26].

As of 2018, according to the UNAIDS country progress report for Pakistan, ever since the Ministry of Health was transferred to the provinces in 2010, the HIV response has struggled due to various factors such as poor coordination among confederal units, the apathy of health authorities, errors in the cases coverage, variabilities in initiatives, inadequate public participation, and willful misconduct by unlicensed and underqualified individuals who purport to provide medical care [27]. When an unexpected HIV outbreak was observed in April 2019 in the small rural community of taluka Ratodero, the present treatment centers did not offer the necessary laboratory testing nor did they have the budget to purchase or import test kits. Therefore, patients were frequently directed to private laboratories whose fees the majority of patients could not afford, leading to them unable to get treated and thus lead to the increase in untreated HIV cases [28].

Furthermore, along with the aforementioned reasons, the pandemic is one of the top contributors for the mismanagement of HIV cases and an increased number of AIDS cases. Pakistan's National AIDS control Program has a total of 240,000 HIV cases that have officially been registered. Only 55.62% of those registered patients were on ART therapy which is provided via the 50 ART centers that currently operate in the country, which is insufficient for such a populous country [16]. Additionally, the pandemic goes on to exacerbate the delays in HIV testing and diagnosis while also causing a hindrance in the proper treatment therapies for the infection. Most of the resources that were previously dedicated to the management of HIV/AIDS have been allocated to the management of the pandemic instead, further causing a break in the proper control of the HIV/AIDS pandemic [16].

On June 7, 2022, the Health Department of Government of Sindh launched the Pre-exposure Prophylaxis Program (PrEP) in Karachi, in collaboration with the United Nations in Pakistan [29]. The program offers community-based PrEP to serodiscordant couples and other target populations [29]. Following its launch event, the ART (antiretroviral therapy) Center staff was trained on effectively providing PrEP services and was linked to outreach workers on the frontline [29]. These workers refer high-risk individuals from the community to ART treatment centers for testing and counselling [29]. In addition, they also provide HIV prevention packages, safe sex education and other resources necessary for behavioral changes [29]. Nai Zindagi, an organization that started as a small residential drug therapy center in Lahore in 1989, has now well-established itself and primarily works with street-based injecting drug users (IDUs), providing them with counselling, rehabilitation, and testing facilities [30]. Moreover, it is also involved in dispensing clean needles in exchange for used or dirty ones to help reduce the risk for contracting HIV [30]. A similar approach was taken by RAAH Foundation in 2016 and has been working since to reduce HIV/AIDS and STDs in Pakistan, focusing on key populations like transgenders, female sex workers, men who sex with men and IDUs [31].

Following the HIV outbreak in Larkana, Sindh in April 2019, the *HIV Communications Project Part 1* was set in motion [32]. The Providence/Boston Center for AIDS Research (CFAR) collaborated with the Jinnah Sindh Medical University Alumni Association of North American (JSMUAANA), the Jinnah Sindh Medical University (JSMU), Association of Physicians of Pakistani Descent of North America (APPNA), and the Association of Pakistani Physicians of New England (APPNE) to make this possible [32]. This project aimed to reduce stigma, train healthcare providers and work with various academic institutions to create awareness regarding the disease and its transmission [33]. A multitude of activities were coordinated under this project to achieve these goals. A total of six webinars were conducted under the supervision of the Project Director, Fizza Gillani, and featured lecturers from Brown University Medical School [33]. These lectures encompassed some imperative HIV related topics such as testing, stigma, coinfections with HIV and role of mental health during HIV treatment [33]. The project coordinators met with the National AIDS Control Program (NACP) in Islamabad to discuss possible collaboration with the Providence/Boston CFAR, training and prevention programs, and other funding opportunities [33]. The team engaged with local academic institutions like NUST (National University of Science and Technology) and Pakistan Institute of Development Economics (PIDE) to work on expanding economic capacity of the disease [33]. Additionally, the team connected with a private school system group that administered over 1500 schools in many rural areas of Pakistan and worked to incorporate a health education module into their curriculum [33]. Antiretroviral drugs are available only in government hospitals [34]. This improves the process of the patient's record keeping and with routine follow-ups, lowers the chances of developing drug resistance [34]. However, patients from farther areas cannot access these treatment facilities [34].

In terms of recommendations, there are a number of ways to reduce the prevalence of HIV in Pakistan. First and foremost, the national government can work closely with provincial governments to focus on increasing general awareness, centers, the accessibility of treatments, and testing kits in urban areas. This is essential as 87% of HIV positive patients were urban dwellers [35]. According to the Join United Nations Programme on HIV and AIDS (UNAIDS), one way in which the national government can act is by reinstating the Ministry of Health to the provinces. This ministry was dissolved in 2010 and thus the HIV response has been lagging due to the inefficiency in coordination with various aspects of federal and provincial governments, inaccuracy of reported HIV cases, inability to engage the community and finally medical malpractice by unqualified individuals [27]. Furthermore, the government can also work with international humanitarian organizations to gather necessary funding to reduce the number of HIV cases within the country. According to the Ministry of Health, the immediate requirement of funds in its national strategic 5-year plan requires $266 million dollars [36]. Thankfully, organizations such as The Global Fund has already provided funding to the country however the country still lacks an approximate $201 million dollars [36]. It would be immensely beneficial if the country can appeal to other organizations or even other country's governments to produce the rest of the funding required to reduce the steadily increasing number of HIV cases and treat the already diagnosed HIV patients.

Another aspect the government can focus on is it's work within its own healthcare system by encouraging healthcare workers or institutions to spread awareness of HIV to the general public as well as crackdown on unqualified individuals within its own system who are contributing to the rising number of HIV cases. An estimated 89.6% of HIV patients are not receiving any forms of treatment [27]. The most significant reasons for this as well as the overall late initiation of HIV treatment from patients have been reported to be the lack of symptoms, the unwillingness to communicate their HIV test findings as well as social stigmas and the fear of prejudice from the patient's own community [16]. Therefore, the government can work with healthcare workers to help spread awareness of HIV, the need for screening, treatment as well as preventative measures that can be taken by individuals. Moreover, healthcare workers need to hold themselves accountable as well as those who aren't qualified to give healthcare treatments as studies report that up to 94% of injections are administered with used injection equipment [13]. This coupled along with the fact that Pakistan itself has a high rate of medical injections, 4.5 per capita per year, it is clear how this can easily lead to a spike in the number of HIV infections via blood donations transfusions [13]. Finally, according to various sources, a large number of HIV patients are injection drug users with some sources suggesting that this subset makes up to 78% of HIV patients [35]. This makes it evident that healthcare institutions would do well to screen any incoming overdosing patients or patients with a history of drug abuse and or drug addiction for HIV and to then treat them appropriately.

## Ethical approval

Not Needed.

## Sources of funding

No financial or material support to report.

## Author contribution

Yumna Salman: Conceived the idea and design, manuscript preparation, manuscript editing, organized references and manuscript review, final approval, and agreeing to the accuracy of the work.

Sean Kaisser Shaeen: Wrote recommendations, manuscript review, final approval, and agreeing to the accuracy of the work.

Malika Saeed Butt: Wrote the introduction, final approval, and agreeing to the accuracy of the work.

Laiba Imran Vohra: Wrote challenges, final approval, and agreeing to the accuracy of the work.

Taleen Hashmi: Wrote efforts, final approval, and agreeing to the accuracy of the work.

## Conflicts of interest

None to Report.

## Consent

Not needed.

## Registration of research studies

1. Name of the registry: N/A.

2. Unique Identifying number or registration ID: N/A.

3. Hyperlink to your specific registration (must be publicly accessible and will be checked): N/A.

## Guarantor

Yumna Salman, Sean Kaisser Shaeen, Malaika Saeed Butt, Laiba Imran Vohra, Taleen Hashmi.

## Abbreviations

ART: Anti Retro-Viral Therapy
HCW: Healthcare workers
UNIAIDS: Joint United Nations Programme on HIV/AIDS
NACP: National AIDS Control Program
APPNA: Association of Physicians of Pakistani Descent of North America
APPNE: Association of Pakistani Physicians of New England
NUST: National University of Science and Technology
PIDE: Pakistan Institute of Development Economics
